# Effect of thermal treatment on the growth, structure and luminescence of nitride-passivated silicon nanoclusters

**DOI:** 10.1186/1556-276X-6-168

**Published:** 2011-02-23

**Authors:** Patrick RJ Wilson, Tyler Roschuk, Kayne Dunn, Elise N Normand, Evgueni Chelomentsev, Othman HY Zalloum, Jacek Wojcik, Peter Mascher

**Affiliations:** 1 Department of Engineering Physics and Centre for Emerging Device Technologies, McMaster University, 1280 Main Street West, Hamilton, Ontario L8S4L7, Canada; 2 Department of Physics and Engineering Physics, University of Saskatchewan, 116 Science Place, Saskatoon, Saskatchewan S7N5E2, Canada

## Abstract

Silicon nanoclusters (Si-ncs) embedded in silicon nitride films have been studied to determine the effects that deposition and processing parameters have on their growth, luminescent properties, and electronic structure. Luminescence was observed from Si-ncs formed in silicon-rich silicon nitride films with a broad range of compositions and grown using three different types of chemical vapour deposition systems. Photoluminescence (PL) experiments revealed broad, tunable emissions with peaks ranging from the near-infrared across the full visible spectrum. The emission energy was highly dependent on the film composition and changed only slightly with annealing temperature and time, which primarily affected the emission intensity. The PL spectra from films annealed for duration of times ranging from 2 s to 2 h at 600 and 800°C indicated a fast initial formation and growth of nanoclusters in the first few seconds of annealing followed by a slow, but steady growth as annealing time was further increased. X-ray absorption near edge structure at the Si K- and L_3,2_-edges exhibited composition-dependent phase separation and structural re-ordering of the Si-ncs and silicon nitride host matrix under different post-deposition annealing conditions and generally supported the trends observed in the PL spectra.

## Introduction

Quantum confinement effects have been found to improve the efficiency of radiative recombination in silicon [1]. In accordance with Heisenberg's uncertainty principle, the spatial confinement of the charge carriers induces a spread in their momenta, allowing for quasi-direct radiative transitions to occur in an indirect bandgap semiconductor. Utilizing these quantum confinement effects, efficient light emission has been achieved from silicon nanoclusters (Si-ncs) formed in a dielectric host matrix. While the properties of this luminescence have been observed to depend on the size of the Si-ncs, difficulties arise in the understanding of these materials from the effects related to the Si-nc/dielectric interface, as well as from the specific physical properties of the dielectric matrix. This situation is further compounded by fabrication-specific issues, where the use of different deposition systems or source gases for the fabrication of Si-nc-containing thin films can alter the observed optical behaviour of the materials, requiring continued research to gain a better understanding of this materials system [2,3].

Forming Si-ncs in a silicon nitride host matrix offers several key advantages over silicon oxide, which was the focus of many early studies [4-9]. Silicon nitride is a promising host matrix candidate since it is a structurally stable dielectric commonly used in microelectronic fabrication processes. Favourable electrical properties resulting from the lower tunnelling barriers allow for better transport of electrons and holes into Si-ncs formed in silicon nitride, making these films better suited for electroluminescent device applications [10]. In addition, Si-ncs coordinated with oxygen atoms are subject to charge trapping related to double-bonds between silicon and oxygen at the interface, which effectively limits the emission from such Si-ncs to energies less than approximately 2 eV, regardless of Si-nc dimensions [11]. Since Si-ncs coordinated with nitrogen atoms do not exhibit the same limitation, emission has been demonstrated to occur at energies across the entire visible spectrum [10,12,13]. The process of forming Si-ncs in silicon nitride is also more favourable due to much lower annealing temperature requirements for bright luminescence compared to silicon oxide films where temperatures must typically exceed 1000°C [14]. In fact, even before annealing, silicon-rich silicon nitride (SRSN) films grown by plasma-enhanced chemical vapour deposition (PECVD) can exhibit efficient luminescence. However, the formation of Si-ncs in SRSN films has been found to occur in a more complex fashion, with formation of both amorphous and crystalline clusters being reported and a strong dependence on both deposition and processing conditions [10,15-17].

In this article, Si-ncs formed in SRSN films deposited with varied compositions using three different chemical vapour deposition (CVD)-based systems are compared and discussed: plasma-enhanced CVD (PECVD), inductively coupled plasma CVD (ICP CVD), and electron cyclotron resonance PECVD (ECR PECVD). Results from these studies have been previously reported in two conference proceedings [18,19]. Most studies to date have employed isochronal annealing steps after deposition to induce diffusion of excess silicon to nucleation sites. Conventionally, this has been done using a quartz tube furnace with an ambient gas of N_2 _or N_2 _+ 5% H_2 _(i.e. forming gas) over 60 min. For consistency, this approach has been taken to provide a good comparison amongst the three deposition systems studied. However, whilst this provides for good comparison amongst the results of various studies, to date there has not been an in-depth isothermal study wherein the annealing is performed over a large time scale ranging from seconds to hours. To address this gap in reported data, in this study, SRSN thin films have been annealed for times ranging from 2 s to 2 h using rapid thermal annealing to provide a basis for investigating the growth process and thermal evolution of these films as well as determining the flexibility of the processing conditions over which such a film could be incorporated into a larger device design.

## Experimental details

In comparing the three CVD systems, SRSN thin films were deposited on n-type (100) Si substrates. The sample compositions were controlled through the variation of the deposition gas flow rates, adjusting the nitrogen source rate while keeping the silicon source rate constant. Unless otherwise stated, all depositions were performed with a substrate heater temperature of 300°C, and the system-specific data for the silicon and nitrogen source gases, radio frequency (RF) power for PECVD and ICP CVD, or microwave (MW) power for ECR PECVD, film thickness, and deposition rate are all listed in Table 1. Post-deposition, the samples were subjected to thermal annealing in a quartz tube furnace for 60 min under either flowing N_2 _or N_2 _+ 5% H_2_. The characteristics of the Si-ncs are strongly dependent on both deposition and processing parameters, as evidenced by variations in their measured luminescent properties and electronic structure. The films studied in the isothermal annealing experiments were deposited by the ECR PECVD system using similar parameters as employed in the system comparison, except that the films in this case were grown to be approximately 3000 Å thick and were deposited using a substrate heater temperature of 350°C (unless otherwise stated). The higher temperature was used since this was generally found to produce SRSN films with increased photoluminescence (PL) intensity for this particular system. For better temporal accuracy, the post-deposition annealing was performed using a Qualiflow Jipelec Jetfirst 100 rapid thermal processor (RTP) rather than a quartz tube furnace. The isothermal study was performed using temperatures of 600 and 800°C with a ramp rate of 25°C/s under flowing N_2 _gas for times ranging from 2 to 7200 s. The emission spectra of the films were measured via room temperature ultraviolet-excited PL using a 17 mW HeCd laser emitting at 325 nm. The complete details of our PL setup have been described elsewhere [20]. Film compositions were measured using Rutherford backscattering spectrometry (RBS) conducted in the Tandetron Accelerator Laboratory at the University of Western Ontario. Finally, X-ray absorption near edge structure (XANES) experiments were performed to obtain information on the electronic structure of the films at the Si K- and L_3,2_-edges. The XANES measurements were conducted on the high resolution spherical grating monochromator (SGM) [21] and variable line spacing plane grating monochromator (VLS PGM) [22] beamlines at the Canadian Light Source synchrotron facility. In these experiments, both the total electron yield (TEY) and total fluorescence yield (FLY) were measured simultaneously, normalized to the incident X-ray intensity (I_0_). These yields provide information over different depths within the sample because of the relative mean free paths of secondary electrons and fluorescence photons at the absorption edges probed. Information on the bulk of the film was provided by the TEY spectra at the Si K-edge and the FLY spectra at the Si L_3,2_-edge.

**Table 1:** System specific details for SRSN thin film depositions

CVD system	Si source gas	N source gas	RF/MW power (W)	Film thickness (Å)	Deposition rate (Å/min)
PECVD	5% SiH_4_/Ar	NH_3_	50	2200-2600	110-130
ICP CVD	30% SiH_4_/Ar	N_2_	300	2400-3000	26-30
ECR PECVD	30% SiH_4_/Ar	10% N_2_/Ar	500	800-1200	53-60

## Results and discussion

### Sample composition

The films produced by each of the three deposition systems for the isochronal annealing experiments covered a broad range of compositions from stoichiometric Si_3_N_4 _to 14 at.% excess silicon content (Si_ex_) relative to stoichiometry. Here, the excess silicon content for substoichiometric silicon nitride films with composition SiN_x _has been defined as:

Si_ex _= Si_at.%_/(Si_at.% _+ N_at.%_) - 3/7 = (1 + *x*)^-1 ^- 3/7.

Film compositions were determined by fitting experimental RBS data from the as-deposited (AD) films with simulated spectra using the SIMNRA software package [23] and all quoted percentages in this study refer to atomic percentages derived from these measurements. Owing to the inherently poor sensitivity of RBS in measuring lower atomic number elements such as nitrogen, the values obtained from the fits have been rounded to the nearest percent, and values measured below 0.5% have been labelled as <1% to account for the uncertainty in the data. The films used in the isothermal annealing experiments were measured to be moderately silicon-rich, having excess silicon contents of 2-3%. Of these films, the one used to study the Si K-edge XANES was deposited at a slightly lower substrate temperature of 300°C, which could have a minor effect on the film's properties. However, for the purposes of this study, the compositions of these films were similar enough to draw qualitative comparisons between the trends observed in the PL and XANES spectra obtained from the different samples.

### Isochronal comparison of deposition systems

**Figure 1:**
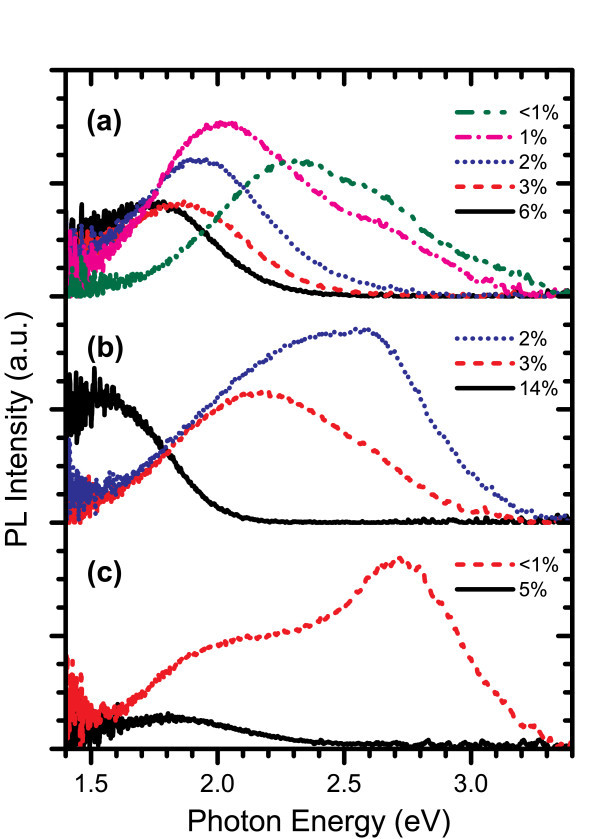
**PL spectra for as-deposited SRSN films grown by (a) PECVD, (b) ECR PECVD, and (c) ICP CVD with their respective excess silicon contents specified in the legend**. As excess silicon content increases, emission shifts to lower energies.

The luminescent properties of the various films were analysed through their room temperature ultraviolet-excited PL spectra. Figure 1 compares the PL spectra from the AD samples from the three systems. Note that the ECR PECVD film with 2% excess silicon content depicted in this figure was grown using a slightly higher substrate heater temperature of 350°C, which may have resulted in higher emission intensity than a film with similar composition deposited at 300°C. Despite the differences in deposition conditions between the systems, similar trends can be observed. Each system produces AD films exhibiting bright PL with emission energies that can be controlled through the full range of the visible and into the near-infrared portion of the electromagnetic spectrum by increasing the excess silicon content in the film. This correlates well with expected quantum confinement effects as Si-ncs increase in size. However, for each system, the emission occurs across a broad range of energies and appears to originate from a combination of quantum confinement effects and defect levels, which have peaks at approximately constant energies independent of the film composition. These defect-related peaks are most prominent in films with low excess silicon content, in which smaller Si-ncs form. As the dimensions of the Si-ncs are reduced, the defect levels become excited and emission through these levels becomes more dominant. The fact that significant PL intensity is observed in the AD films indicates that Si-ncs are formed within SRSN films without the assistance of annealing. This is different from what occurs in silicon-rich silicon oxide (SRSO) films where cluster formation and resulting luminescence occur only after high temperature annealing [14]. The PL intensity of the SRSN films in this study has been qualitatively described as 'bright', which is a rather arbitrary term. Since quantitative measurements of emission intensity have yet to be performed, the term bright is qualified here as PL that is easily visible under typical room lighting conditions.

**Figure 2:**
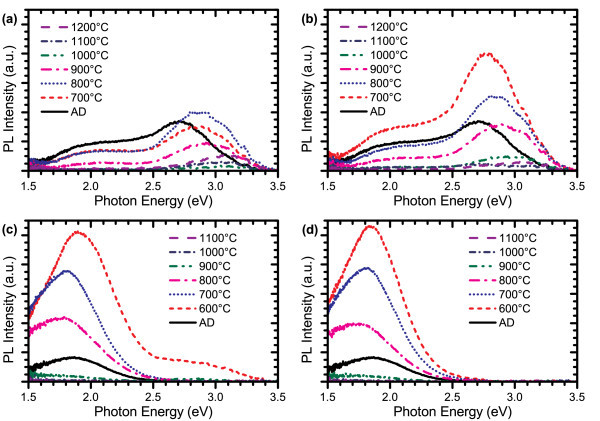
**PL spectra for films annealed for 60 min in a quartz tube furnace**. Shown are an ICP CVD film (Si_ex _< 1%) annealed in **(a) **N_2_, **(b) **N_2 _+ 5% H_2 _and a PECVD film (Si_ex _= 3%) annealed in **(c) **N_2_, and **(d) **N_2 _+ 5% H_2_.

The effects of annealing a PECVD film with moderately high excess silicon content and an ICP CVD film with low excess silicon content using different ambient gases are compared in Figure 2. In general, the emission spectra for samples with higher excess silicon content tend to red-shift slightly as the annealing temperature is increased, whereas lower excess silicon content samples exhibit a slight blue-shift. In samples containing intermediate levels of excess silicon content, the PL peaks have also been observed to blue-shift relative to the AD spectra at low temperatures and red-shift as the annealing temperature is further increased. There appear to be at least two competing mechanisms in the Si-nc growth dynamics related to the growth of existing Si-ncs due to diffusion of silicon atoms in the film and the formation and subsequent growth of new Si-ncs at nucleation sites. The red-shifting resulting from Si-nc growth is much smaller than that observed in SRSO films, but this can be explained by the more diffusion-inhibiting structure of the silicon nitride matrix relative to the silicon oxide matrix [24]. It is also possible that energy may be transferred between smaller and larger Si-ncs, which affects the observed PL spectra. In all of the samples, the most intense emission consistently occurred when annealing was performed at 800°C or below, with peak intensities being observed at lower temperatures for higher silicon content samples. The reason for the decay in PL intensity at higher temperatures is unknown at this time since (a) Si-ncs are still present in TEM images (not shown) and X-ray absorption spectra of these films and (b) the Si-ncs have not grown beyond the quantum confinement regime because of the inhibitive nature of the nitride matrix. As the decay in luminescence does not appear to relate to structural changes in the Si-nc, this suggests that it results from changes in the host nitride matrix or with the interface passivation. Such effects could arise from the strain induced on the Si-ncs by the nitride matrix or a re-ordering of the nitride matrix structure at the Si-nc interface such that non-radiative recombination pathways become available. However, further investigation is required to accurately attribute the source of this phenomenon.

Hydrogen passivation of dangling bonds at the Si-nc interface is also observed to play a significant role in improving the PL efficiency. The use of N_2 _+ 5% H_2 _rather than pure N_2 _as an ambient gas in the annealing process significantly improves the emission intensity in the ICP CVD- and ECR PECVD-deposited films. This enhancement is not observed in the PECVD-deposited films, which may be because this system uses NH_3 _as a nitrogen source. Higher concentrations of hydrogen may remain in the film after dissociating from the NH_3 _gas molecules during the CVD reaction process. Having increased levels of hydrogen in the AD PECVD films could be very beneficial when considering incorporating these types of luminescent films into a larger scale design process, such as for electroluminescent and integrated circuit device processing, provided it does not reduce the quality of the film through increased porosity or the effects of out-gassing. Low temperature rapid thermal annealing is preferable in such cases due to the shorter timescale and reduced thermal budget, providing better compatibility with other materials, structures, or processes. Lower temperatures with shorter anneals become particularly important for avoiding the diffusion of metals from contacts, and potentially reducing the number of design steps required compared to the typically longer quartz tube furnace annealing. The effects of the annealing time on the growth, structure and luminescence of SRSN films are addressed in 'Isothermal anneals at 600°C' and 'Isothermal anneals at 800°C' below.

**Figure 3:**
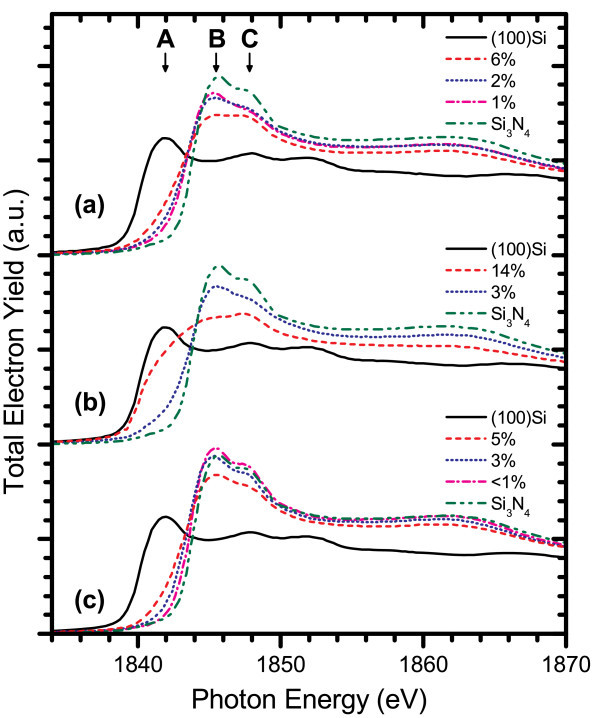
**TEY-XANES spectra for (a) PECVD, (b) ECR PECVD, and (c) ICP CVD AD films at the Si K-edge**. *A*, *B*, and *C *indicate the peak positions for Si-Si, Si-N, and Si-O resonances, respectively. The percentages in the legend refer to the excess silicon content of the SRSN films.

**Figure 4:**
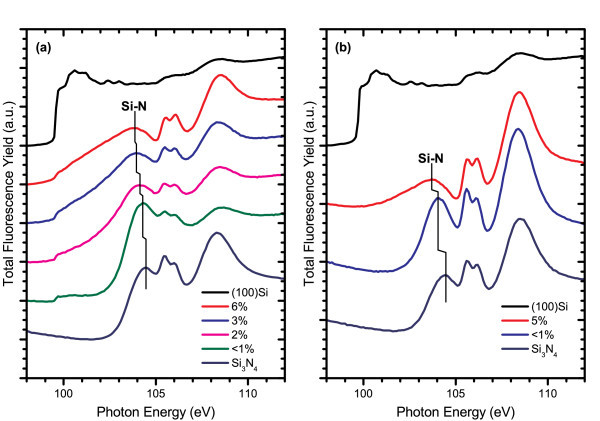
**FLY-XANES spectra for as-deposited (a) PECVD and (b) ICP CVD films at the Si L_3,2_-edge**. The spectra are offset by a constant value in the order they are listed in the legend, in which the excess silicon content of the SRSN films is specified as a percentage. The Si-N resonance peak shifts to lower energies in films with higher excess silicon content.

The electronic structure was probed through X-ray absorption near edge structure experiments at the silicon K- and L_3,2_-edges, where differences in structure within the films can be identified by shifts in their spectral features [25-29]. The XANES measurements performed at the silicon K-edge for AD films from each system are shown in Figure 3, which reveal common trends in the Si-nc structure. The spectra of the ICP CVD films were measured from 2-μm-thick films, much larger than the information depth at either absorption edge [30], to ensure that the substrate would not contribute to the TEY or FLY. However, through further experiments, it has since been found that film thicknesses greater than 1500 Å are sufficient not to exhibit substrate effects in the TEY data at the Si K-edge, or either the TEY or FLY data at the Si L_3,2_-edge. A low doped, n-type (100) silicon wafer was used as a crystalline silicon reference for all of the XANES experiments, and the Si_3_N_4 _reference sample was an AD ICP CVD film with stoichiometric composition. As the silicon content is increased in the films, the absorption edge shifts to lower energies because of the increase of the Si-Si resonance peak at 1842 eV and reduction of the peak related to Si-N bonding located at 1845.5 eV. The weak Si-O peak at 1848 eV in the crystalline silicon reference spectrum arises from the native oxide layer formed at the silicon surface while any Si-O signal exhibited by the SRSN films originates from oxygen contamination at the surface of the film and should not be taken as an indication of Si-O bonding within the bulk of these films. Figure 4 compares the silicon L_3,2_-edge spectra for PECVD and ICP CVD AD films. Both sets of films follow similar trends, with the Si-N resonance peak ranging between 103.8 to 104.5 eV as it shifts to lower energies and broadens at higher excess silicon concentrations. However, the PECVD films have a well-defined Si-Si absorption edge at 99.7 eV, which is absent in the ICP CVD-deposited films. The prominence of the absorption edge in PECVD films could be attributed to a difference in the Si-nc structure or the generation of a greater number of nucleation sites for Si-nc formation resulting from the dissociation of hydrogen from the NH_3 _process gas. Unfortunately, the ECR PECVD films were too thin to avoid a large background signal from the silicon substrate at these energies, and so they have not been included in any of the Si L_3,2_-edge comparisons.

**Figure 5:**
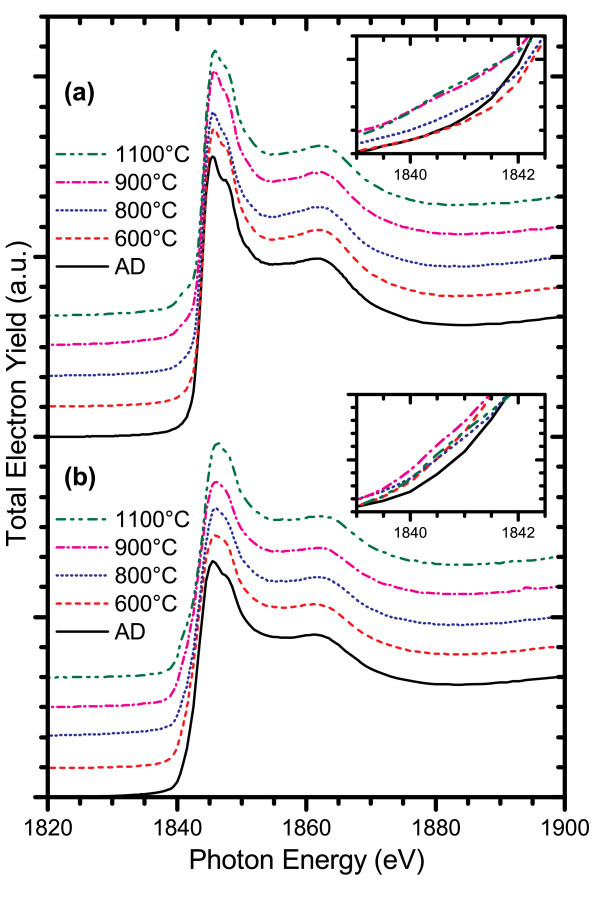
**TEY-XANES spectra at the Si K-edge for (a) low (Si**_**ex **_**< 1%) and (b) high (Si**_**ex **_**= 5%) excess silicon content films deposited by the ICP CVD system and annealed in a quartz tube furnace under N**_**2 **_**+ 5% H**_**2 **_**ambient gas**. The insets included with each plot show a magnified view of the Si-Si absorption edge with the offset between spectra removed. A Si-Si resonance shoulder onsets at temperatures as low as 900°C in the low Si content film and 600°C in the high Si content film.

**Figure 6:**
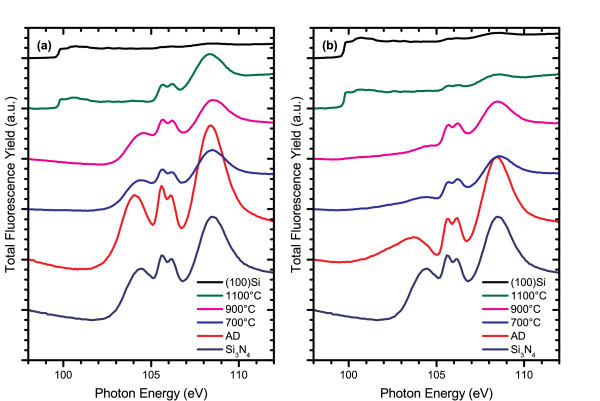
**FLY-XANES spectra at the Si L**_**3,2**_**-edge for (a) low (Si**_**ex **_**< 1%) and (b) high (Si**_**ex **_**= 5%) excess silicon content films deposited by the ICP CVD system and annealed in a quartz tube furnace under flowing N**_**2 **_**+ 5% H**_**2 **_**gas**. The spectra are offset by a constant value in the order they are listed in the legend, and the (100)Si spectra are normalized to the Si-Si absorption edge step in the 1100°C spectra for better comparison.

Figures 5 and 6 show the changes in the Si K- and L_3,2_-edge XANES spectra for two ICP CVD grown films, one with low excess silicon content (Si_ex _< 1%) and the other with high excess silicon content (Si_ex _= 5%), as the annealing temperature is increased. At temperatures of 900°C and above, films with low excess silicon concentration develop a shoulder at the Si-Si bonding energy of 1842 eV, suggesting a change in the Si-nc structure and increased phase separation in these films. The position of the Si-N resonance peak shifts to higher energies, from 1845.5 to 1846 eV, and increases in magnitude as the annealing temperature is increased. At the silicon L_3,2_-edge, the Si-Si absorption edge at 99.7 eV is suppressed, and details of the Si clustering are not observed while the nitride matrix undergoes a clear change in structure up to 1100°C when the nitride matrix appears to break down. In films with high excess silicon content, the onset of the Si-Si shoulder in the silicon K-edge spectra occurs at temperatures as low as 600°C. This indicates that the phase separation and Si-nc formation are not solely dependent on the nitride host matrix and are instead strongly influenced by the composition of the deposited film. Changes to the Si-N peaks in the silicon K- and L_3,2_-edge spectra once again reflect structural changes in the nitride matrix. At the silicon L_3,2_-edge, the details of Si-Si bonding are also suppressed in these films until 1100°C where the nitride matrix breaks down.

**Figure 7:**
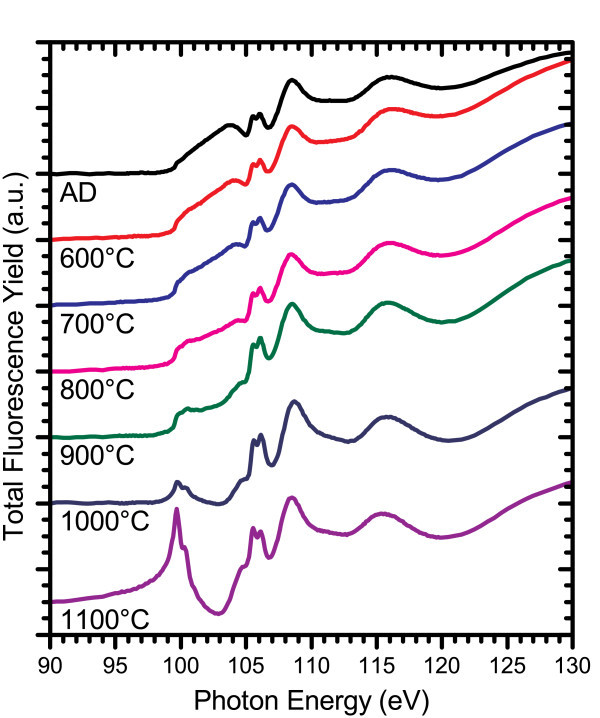
**FLY-XANES spectra at the Si L**_**3,2**_**-edge for a high excess silicon content PECVD film (Si**_**ex **_**= 6%) annealed in a quartz tube furnace under N**_**2 **_**ambient gas**. The offset spectra are labelled underneath.

Analysis at the silicon L_3,2_-edge is hindered by substantial distortion of the FLY signal due to either self-absorption effects, which intensify as the film density increases with higher annealing temperatures, or augmentation of X-ray scattering resulting from voids formed within the film [31]. Preliminary results from positron annihilation spectroscopy experiments suggest that void formation is at least partially responsible for the distortion observed, but it remains to be established as a full investigation of this effect is still underway. The distortion is most prominent in high excess silicon content films deposited by the PECVD system, although it is observed to some degree in all of the SRSN films measured at the Si L_3,2_-edge. An example of this effect is shown in Figure 7. As the annealing temperature is increased, a dip grows in the FLY at energies between the Si-Si absorption edge and the higher energy side of the Si-N resonance peak. A full account of this effect is a non-trivial challenge yet to be corrected for this data, which, however, is certainly necessary to gain accurate and specific information on the changes in the silicon nitride host matrix.

### Isothermal anneals at 600°C

**Figure 8:**
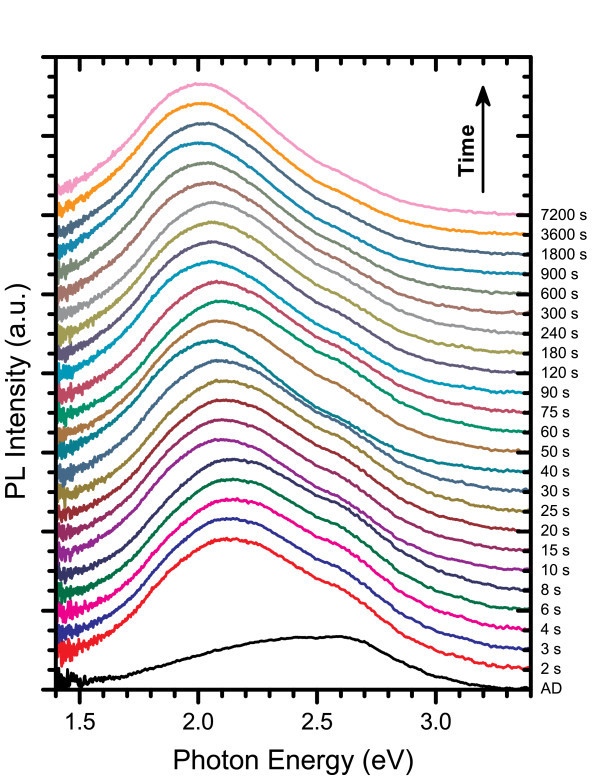
**PL spectra for films with Si**_**ex **_**= 3% annealed at 600°C**. The annealed spectra are renormalized to have equal peak heights and offset in order of increased annealing time to clearly show the shifting in peak PL energy that occurred with annealing.

As described previously, in the case of isochronal annealing for 60 min in a quartz tube furnace, the PL of SRSN films with moderate-to-high excess silicon content tends to shift towards lower energies as the annealing temperature increases. Such a shift is in agreement with theory for quantum confinement effects corresponding to the growth of Si-ncs where the bandgap energy is proportional to the inverse square of the nanocluster diameter. Figure 8 shows the PL spectra for a film with 3% excess Si content annealed at 600°C for time intervals ranging from a mere 2 s to 2 h. In this figure, the annealed PL spectra were renormalized to have the same peak height to aid in comparing changes in emission energies while the AD spectra was renormalized to maintain its relative intensity compared to the 2 s anneal. Each spectrum consisted of a main peak that shifted to lower energies as the annealing time increased, and a higher energy shoulder that was most prominent in the AD film, which diminished as the annealing time increased. There was an abrupt red-shift in peak emission energy from 2.58 eV in the AD film to 2.13 eV after only 2 s of annealing along with a large increase in intensity. As the annealing time was increased further, the PL peak continued to shift towards lower energies, but these changes were relatively small compared to the initial shift. This indicates that Si-ncs form and begin to grow very rapidly through a transient diffusion of excess silicon.

**Figure 9:**
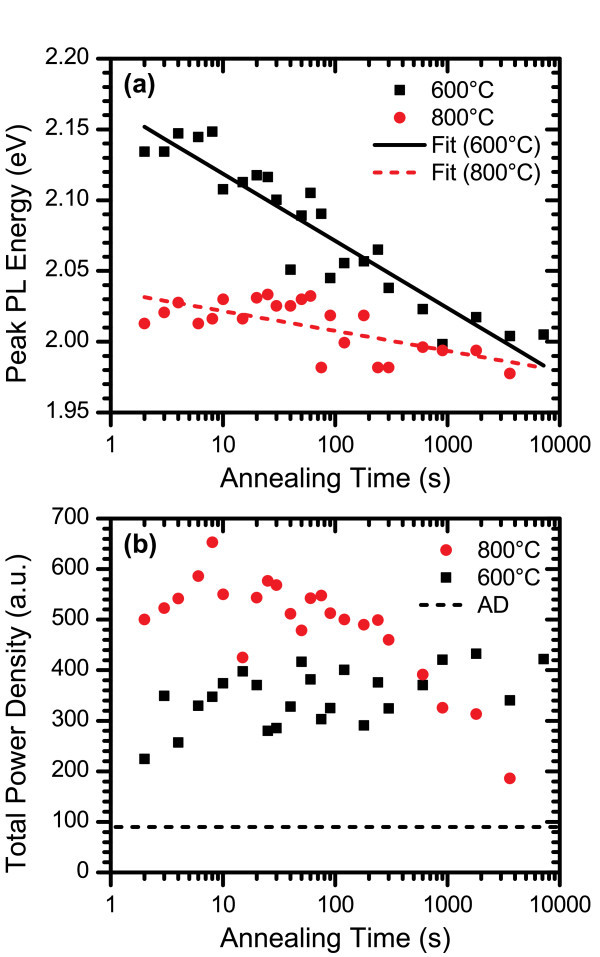
**PL characteristics of films with Si**_**ex **_**= 3% annealed at 600 and 800°C**. The plots depict **(a) **the peak PL energy and **(b) **the total power density of films annealed for times ranging from 2 s to 2 h under flowing N_2 _ambient gas. Logarithmic fit lines are included in **(a) **to emphasize the trend of peak PL energy shifting to lower energies with longer annealing times and are not intended to represent a model.

The peak emission energies of the annealed spectra are shown on a semilog plot in Figure 9a. The peak PL energy was determined by applying a Savitzky-Golay smoothing filter to remove the effects of noise without distorting the shape of the spectra and locating the energy at which the peak PL intensity occurred. There is a clear and steady shift from approximately 2.15 eV for the very short anneals towards 2.00 eV for annealing times approaching 2 h in length. The trend is characterized in the diagram by a logarithmic fit of the data points. The high energy shoulder in the PL spectra can be attributed to one of the silicon nitride inter-bandgap defect levels [32], which was annealed out as the length of annealing time increased. Figure 9b shows a semilog plot of the total power density of the annealed films as a function of annealing time with a dashed line representing the total power density of the AD film. Annealing caused a sharp increase in the PL intensity even at the shortest annealing times. Following this sudden increase, the total power density for the 600°C anneals continued to improve as the annealing time increased up to 2 h, albeit at a much slower rate.

**Figure 10:**
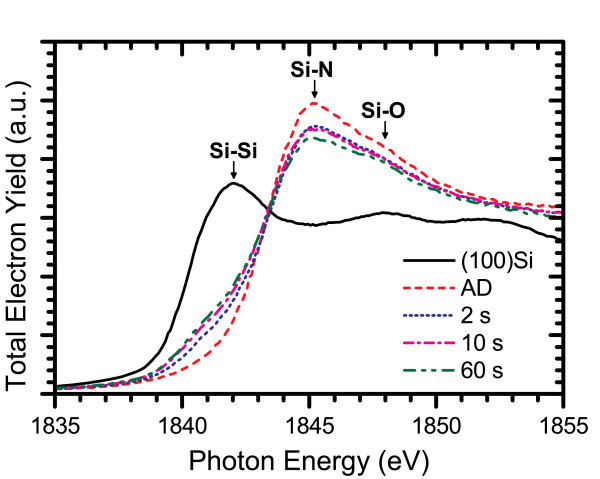
**TEY-XANES spectra at the Si K-edge for a film with Si**_**ex **_**= 2% annealed for different times at 600°C**.

**Figure 11:**
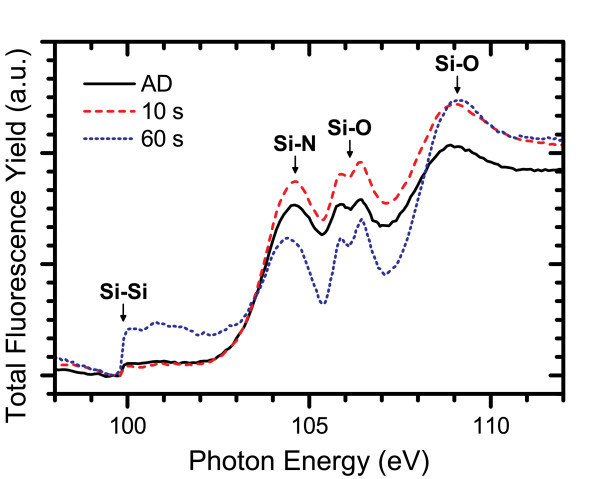
**FLY-XANES spectra at the Si L**_**3,2**_**-edge for a film with Si**_**ex **_**= 3% annealed for different times at 600°C**.

XANES measurements provided insight on the structural ordering of the Si-ncs and the silicon nitride host matrix. Several spectra measured at the Si K- and L_3,2_-edges are shown in Figures 10 and 11, respectively. At the Si K-edge, a gradual increase in Si-Si bonding was observed in a 3% excess Si content film with increasing annealing time corresponding to larger Si-ncs and increased phase separation. Also, there was a large increase in the Si-Si bonding resonance over the AD spectrum even at very short annealing times. Large restructuring of the silicon nitride host matrix was also observed on the same time scale as evidenced by the significant changes in the Si-N bonding resonance over the course of annealing. Similar changes were obtained at the Si L_3,2_-edge for a film with 2% excess Si content, where the Si-Si absorption edge becomes very large after the 60 s anneal and significant changes in both the peak energy and the magnitude of the Si-N resonance are observed over the timescale studied. Combined with the large changes measured in the PL spectra for annealing times on the order of seconds, these results suggest that Si-ncs form much more rapidly than has been conventionally believed and it is likely the result of a fast transient diffusion mechanism for excess silicon in a silicon nitride film.

### Isothermal anneals at 800°C

**Figure 12:**
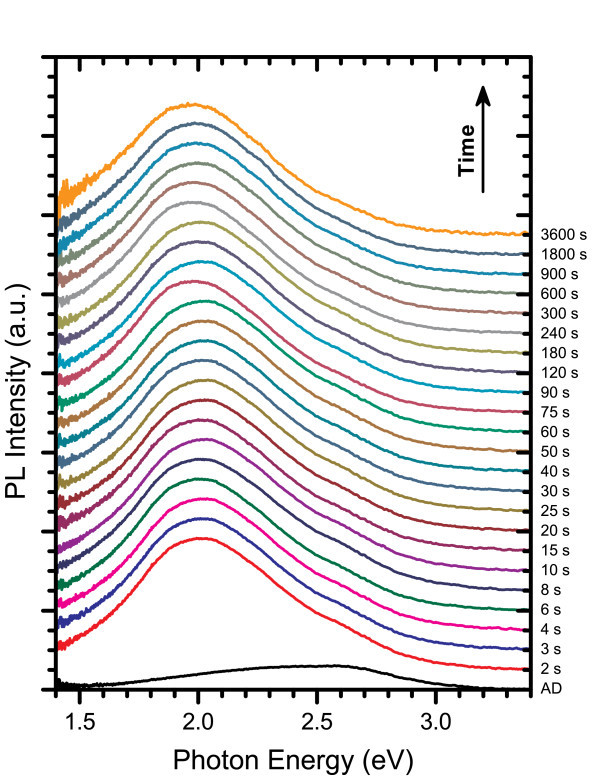
**PL spectra for Si**_**ex **_**= 3% films annealed at 800°C**. The annealed spectra are renormalized to have equal peak heights and offset in order of increased annealing time.

The PL spectra for the film with 3% excess silicon content annealed at 800°C exhibited the same features as those of the 600°C annealed films as can be seen in Figure 12. As in Figure 8, the annealed spectra have been renormalized so that they have the same peak intensity while the AD spectrum has been renormalized so that it maintained its relative intensity with the 2 s anneal. In this case, the AD peak appears smaller than in Figure 8 due to the relatively large PL intensity of the 2-s-annealed film at 800°C compared with its 600°C counterpart. At 800°C, there was still a main peak that red-shifted with longer annealing times and a high energy shoulder that was less pronounced than at the lower temperature and nearly disappeared at the longer annealing times. The peak PL energy is plotted in Figure 9a, which illustrates that the initial abrupt energy shift upon annealing is much larger than for the 600°C anneals and even exceeds the shift observed for all but the longest anneals measured at this temperature. However, for longer anneals, the peak PL energy shifted at a much slower rate than at 600°C. This was likely due to the reduction of excess silicon in the film within close proximity of a Si-nc that has not already been incorporated into the structure and the larger number of additional Si atoms required for continuing to increase the diameter of a Si-nc as it grows. The total power density profile shown in Figure 9b shows some interesting differences to those observed after the 600°C anneal. At 800°C, there was a very large increase in the emission intensity after just 2 s of annealing, which also far exceeded the total power densities measured for any of the 600°C anneals. While an overall increase in total power density was observed at 600°C over the range of annealing times studied, an intensity peak was observed between 6 and 30 s at the higher temperature, followed by a steady decline, eventually dropping below the 600°C value at the 900 s mark. This decline may indicate Ostwald ripening or structural changes in the silicon nitride host matrix.

**Figure 13:**
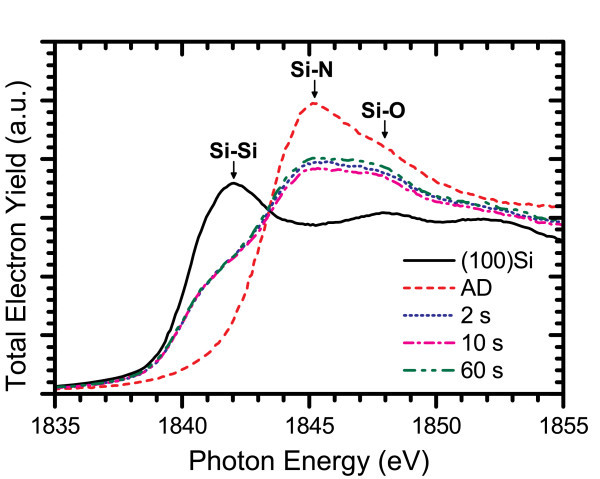
**TEY-XANES spectra at the Si K-edge for the Si**_**ex **_**= 2% film annealed for different times at 800°C**.

The occurrence of Ostwald ripening and silicon nitride structural reordering are evidenced by the Si K-edge XANES spectra for the 2% excess Si content film shown in Figure 13. These spectra exhibit a large increase in the Si-Si resonance after just 2 s of annealing but no noticeable change as the annealing time is extended, suggesting that further increases in Si-nc size are due to larger nanoclusters growing at the expense of smaller ones. At the same time, large changes were observed in the Si-N resonance, which include a significant increase between the 10 and 60 s anneals.

It is probable that the decay in PL intensity observed for longer anneals at 800°C will occur after annealing for a minimum time at higher temperatures as well. If this assumption is true and the onset of decay occurs at earlier times as the temperature is increased, then this phenomenon may be linked to the decrease in PL intensity observed in SRSN films annealed for 60 min in a quartz tube furnace at temperatures above 700 or 800°C. Incidentally, as shown in Figure 9b, the 60 min mark resides in the time interval where the 800°C annealed films became less intense than the 600°C annealed films.

## Conclusions

We have demonstrated that bright luminescence can be attained from Si-ncs formed in SRSN thin films deposited by PECVD, ICP CVD and ECR PECVD using different combinations of source gases. Each system produced films with highly tunable luminescence through adjustment of the process gas flow rates. Post-deposition annealing only had a minor impact on the peak PL energy, but the annealing temperature and ambient gas strongly affected the PL intensity. For 60 min anneals in a quartz tube furnace, the best results were achieved at low temperatures under flowing N_2 _+ 5% H_2 _gas. Hydrogen appeared to play an important role in enhancing luminescence from SRSN films. Much of this may be attributed to hydrogen passivation of dangling bonds at the Si-nc surfaces, but XANES spectra at the Si K- and L_3,2_-edge also indicated that hydrogen incorporated within the AD film may increase the number of nucleation sites for Si-nc formation. In addition, the XANES spectra provided evidence of composition-dependent phase separation and structural re-ordering of both the Si-ncs and the nitride host matrix upon annealing. Unfortunately, self-absorption or photon scattering from void formation in the film obscures the Si-Si and Si-N resonance peaks at the Si L_3,2_-edge, and a full account of this effect has yet to be realized. This obstacle must be addressed before realistic information about the Si-nc and nitride host matrix structures could be derived from such spectra.

Expanding upon the results obtained from the isochronal annealing experiments, an extended series of time-varied anneals of SRSN films was performed at 600 and 800°C using a rapid thermal processor. Based on these experiments, it has been shown that the luminescent and structural properties were in accordance with those expected from theory if emission occurs through quantum confinement effects. The PL peak steadily shifted to lower energy as the annealing time was increased at both temperatures correspondingly with increasing diameter of Si-ncs. Further, the peak shifting occurred more slowly as it became lower in energy, which could be expected since a greater number of additional Si atoms must be added to further increase the Si-nc diameter and make the nanoclusters grow larger in size. Remarkably, the Si-ncs appeared to form and grow very rapidly, with large, abrupt shifts in peak PL intensities of 0.45 and 0.57 eV relative to the AD film after only 2 s of annealing at 600 and 800°C, respectively. The apparent fast growth was indicative of a fast transient diffusion mechanism for excess silicon within SRSN films. The intensity of the annealed films was also an interesting fact in that the total power density showed an increasing trend with longer annealing times over the time period studied for the lower temperature anneals. However, the higher temperature anneals peaked in total power density after 6-30 s of annealing before steadily decaying with longer times. The decay in total power density observed in the higher temperature data was attributed to the Si-ncs undergoing Ostwald ripening and restructuring in the silicon nitride host matrix. XANES spectra at the Si K- and L_3,2_-edges, which revealed a steady increase in the Si-Si bonding resonance in the 600°C films following an abrupt increase after 2 s of annealing, support the proposed growth model. These spectra also exhibited large changes in the Si-N resonance as the films were annealed. At 800°C, a much larger increase in the Si-Si resonance was observed after 2 s of annealing, but this peak did not grow noticeably larger as the annealing time was further increased, which supports the possibility of Ostwald ripening. There was also a large change in the Si-N resonance between 10 and 60 s of annealing, which suggested that the decay in luminescence intensity observed at longer annealing times could also be related to restructuring of the silicon nitride matrix.

## Abbreviations

AD: as-deposited; CVD: chemical vapour deposition; ECR PECVD: electron cyclotron resonance PECVD; FLY: total fluorescence yield; I_0_: incident X-ray intensity; ICP CVD: inductively coupled plasma CVD; MW: microwave; PECVD: plasma-enhanced CVD; PL: photoluminescence; RBS: Rutherford backscattering spectrometry; RF: radio frequency; RTP: rapid thermal processor; SGM: spherical grating monochromator; SRSN: silicon-rich silicon nitride; SRSO: silicon-rich silicon oxide; TEY: total electron yield; VLS PGM: variable line spacing plane grating monochromator; XANES: X-ray absorption near edge structure.

## Competing interests

The authors declare that they have no competing interests.

## Authors' contributions

PRJW carried out or participated in all aspects of both the isochronal and isothermal studies and drafted the manuscript. TR participated in all aspects of the isochronal study as well as the acquisition of XANES data in the isothermal study. KD participated in the acquisition and analysis of RBS data as well as the acquisition of XANES data. ENN participated in the acquisition of XANES data. EC participated in the deposition of the ECR PECVD films. OHYZ participated in the acquisition of PL spectra. JW participated in the deposition of ICP CVD and ECR PECVD films in this study. PM conceived of the study and participated in its design and coordination. All authors read and approved the final manuscript.
